# Supplementary material for: Dynalign II: common secondary structure prediction for RNA homologs with domain insertions
Source: Nucleic Acids Res. 2014 Nov 21;42(22):13939–48. doi: 10.1093/nar/gku1172 (PMC4267632; doi:10.1093/nar/gku1172)
Supplement: SUPPLEMENTARY DATA [file supp_gku1172_nar-02021-z-2014-File012.zip › manual/javadoc/RNAstructure_java_interface/source/startup/RNAstructureBackendCalculator.html]

RNAstructureBackendCalculator


JavaScript is disabled on your browser.


- Overview
- Package
- Class
- Tree
- Index
- Help

- Prev Class
- Next Class

- Frames
- No Frames

- All Classes

- Summary:
- Nested |
- Field |
- Constr |
- Method

- Detail:
- Field |
- Constr |
- Method


RNAstructure\_java\_interface.source.startup

## Class RNAstructureBackendCalculator

- java.lang.Object
- - RNAstructure\_java\_interface.source.startup.RNAstructureBackendCalculator

- ---

    

  ```
  public class RNAstructureBackendCalculator
  extends Object
  ```

- - ### Field Summary

    Fields

    | Modifier and Type | Field and Description |
    | --- | --- |
    | `protected boolean` | `swigCMemOwn` |
    | `private long` | `swigCPtr` |
  - ### Constructor Summary

    Constructors

    | Modifier | Constructor and Description |
    | --- | --- |
    |  | `RNAstructureBackendCalculator()` |
    | `protected` | `RNAstructureBackendCalculator(long cPtr, boolean cMemoryOwn)` |
  - ### Method Summary

    Methods

    | Modifier and Type | Method and Description |
    | --- | --- |
    | `void` | `activateMultilign()` |
    | `void` | `activateTurboFold()` |
    | `void` | `addMultilignTuple(String seqFile, String ctFile)` |
    | `void` | `addTurboFoldTuple(String seqFile, String ctFile)` |
    | `String` | `buildAllSubDataStructure(String file, boolean isRNA)` |
    | `String` | `buildBifoldDataStructure(String file1, String file2, boolean isRNA)` |
    | `String` | `buildBipartitionDataStructure(String file1, String file2, boolean isRNA)` |
    | `String` | `buildDynalignDataStructure(String file1, String file2, boolean isRNA)` |
    | `String` | `buildEfn2DataStructure(String file, boolean isRNA)` |
    | `String` | `buildFoldDataStructure(String file, boolean isRNA)` |
    | `String` | `buildMaxExpectDataStructure(String file)` |
    | `String` | `buildOligoScreenDataStructure()` |
    | `String` | `buildOligoWalkDataStructure(String file, boolean isRNA)` |
    | `String` | `buildPartitionDataStructure(String file, boolean isRNA)` |
    | `String` | `buildProbKnotDataStructure(String file)` |
    | `String` | `buildRefoldDynalignDataStructure(String file)` |
    | `String` | `buildRefoldSingleDataStructure(String file)` |
    | `String` | `buildRemovePseudoknotsDataStructure(String file, boolean isRNA)` |
    | `String` | `buildStochasticDataStructure(String file)` |
    | `boolean` | `canFoldOligoOligo(int index)` |
    | `boolean` | `canFoldOligoSelf(int index)` |
    | `void` | `clearDynalignAlignmentConstraints()` |
    | `void` | `clearFoldingConstraints(int strand)` |
    | `void` | `delete()` |
    | `void` | `deleteMultilignTuple(long index)` |
    | `void` | `deleteTurboFoldTuple(long index)` |
    | `int` | `determineOligoMaximum(int length)` |
    | `protected void` | `finalize()` |
    | `void` | `foldOligo(String sequence, int index, boolean bimolecular, boolean isRNA, String file)` |
    | `String` | `getAllOligoData(int height)` |
    | `protected static long` | `getCPtr(RNAstructureBackendCalculator obj)` |
    | `String` | `getDisplayedOligo(int index)` |
    | `String` | `getDynalignAlignmentConstraints()` |
    | `int` | `getDynalignAlignmentWindowSize()` |
    | `int` | `getDynalignStructureWindowSize()` |
    | `String` | `getFoldingConstraints(int strand)` |
    | `int` | `getFoldWindowSize()` |
    | `int` | `getGraphRegionBegin()` |
    | `int` | `getGraphRegionEnd()` |
    | `int` | `getMaxConstraintIndex(int strand)` |
    | `int` | `getMaxLoop()` |
    | `int` | `getMaxPair()` |
    | `int` | `getMostStableOligo()` |
    | `String` | `getMultilignCT(int index)` |
    | `int` | `getMultilignMaxPairs()` |
    | `String` | `getMultilignSequenceSetData()` |
    | `int` | `getNumMultilignSequences()` |
    | `int` | `getNumTurboFoldSequences()` |
    | `String` | `getOligoLabelData(int index)` |
    | `int` | `getOligoTargetLength()` |
    | `String` | `getOligoTargetSequence()` |
    | `int` | `getProgressNumber()` |
    | `int` | `getRefoldWindowSize()` |
    | `String` | `getSequenceComment()` |
    | `String` | `getSequenceData()` |
    | `String` | `getSequenceTitle()` |
    | `String` | `getStructureType()` |
    | `double` | `getSuboptimalAbsoluteDiff()` |
    | `float` | `getSuboptimalPercentDiff()` |
    | `double` | `getTemperature()` |
    | `String` | `getTurboFoldCT(int index)` |
    | `String` | `getTurboFoldSaveFile(int index)` |
    | `String` | `getTurboFoldSequenceSetData()` |
    | `String` | `readDynalignAlignmentConstraintsFile(String file)` |
    | `String` | `readFoldingConstraintsFile(String file, int strand)` |
    | `void` | `readSequenceData(String file)` |
    | `String` | `runAllSub(String ct, float percent, double absolute)` |
    | `String` | `runBifold(String ctFile, float percent, int maxStructures, int windowSize, boolean saveFile, boolean intraForbidden)` |
    | `String` | `runBipartition(String pfsFile)` |
    | `String` | `runDynalign(String ctFile1, String ctFile2, String saveFile, String alignFile, double percent, int structures, int windowStr, int windowAli, float gap, boolean isInsert)` |
    | `String` | `runDynalignRefold(String ctFile1, String ctFile2, String alignFile, int percent, int structures, int windowStr, int windowAli)` |
    | `String` | `runEfn2(String outFile, boolean writeDetails)` |
    | `String` | `runFold(String ctFile, int percent, int structures, int window, boolean save)` |
    | `String` | `runMaxExpect(String ctFile, double score, int structures, int window, double gamma)` |
    | `String` | `runMultilign(int percent, int maxStructures, int bpWindow, int alignWindow, double gap, boolean insert, double maxDsvChange, int maxPairs, int cycles, String alignFile, boolean saveFiles, boolean isRNA)` |
    | `String` | `runOligoScreen(String in, String out, boolean isRNA)` |
    | `String` | `runOligoWalk(String report, int mode, String chemistry, boolean suboptimal, int length, int amount, String unit, int start, int stop)` |
    | `String` | `runPartition(String pfsFile)` |
    | `String` | `runPseudoknotPrediction(String ctFile, int iterations, int helix)` |
    | `String` | `runPseudoknotRemoval(String ctFile, boolean minimize)` |
    | `String` | `runRefold(String ctFile, int percent, int structures, int window)` |
    | `String` | `runStochastic(String outFile, int ensemble, int seed)` |
    | `String` | `runTurboFoldMaximumExpectedAccuracy(double turboGamma, int turboIterations, double percent, int structures, int window, double meaGamma)` |
    | `String` | `runTurboFoldPseudoknot(double turboGamma, int turboIterations, int pkIterations, int helix)` |
    | `String` | `runTurboFoldThreshold(double turboGamma, int turboIterations, double threshold)` |
    | `String` | `setCleavedNucleotide(int nuc, int strand)` |
    | `String` | `setDoubleStrandedNucleotide(int nuc, int strand)` |
    | `String` | `setDynalignAlignmentConstraint(int nuc1, int nuc2)` |
    | `String` | `setForcedHelix(int nuc1, int nuc2, int length, int strand)` |
    | `void` | `setMaxLoop(int newLoop)` |
    | `void` | `setMaxPair(int newPair)` |
    | `String` | `setModifiedNucleotide(int nuc, int strand)` |
    | `String` | `setProhibitedHelix(int nuc1, int nuc2, int length, int strand)` |
    | `void` | `setSequenceComment(String comment)` |
    | `void` | `setSequenceData(String data)` |
    | `void` | `setSequenceTitle(String title)` |
    | `void` | `setSHAPEFile(String file)` |
    | `void` | `setSHAPEParam1(double value)` |
    | `void` | `setSHAPEParam2(double value)` |
    | `void` | `setSHAPEType(boolean isEnergy)` |
    | `String` | `setSingleStrandedNucleotide(int nuc, int strand)` |
    | `void` | `setTemperature(double newTemp)` |
    | `void` | `writeDynalignAlignmentConstraintsFile(String file)` |
    | `String` | `writeFastaFile(String file)` |
    | `String` | `writeFoldingConstraintsFile(String file, int strand)` |
    | `String` | `writeSequenceFile(String file)` |

    - ### Methods inherited from class java.lang.Object

      `clone, equals, getClass, hashCode, notify, notifyAll, toString, wait, wait, wait`

- - ### Field Detail


    - #### swigCMemOwn

      ```
      protected boolean swigCMemOwn
      ```


    - #### swigCPtr

      ```
      private long swigCPtr
      ```
  - ### Constructor Detail


    - #### RNAstructureBackendCalculator

      ```
      public RNAstructureBackendCalculator()
      ```


    - #### RNAstructureBackendCalculator

      ```
      protected RNAstructureBackendCalculator(long cPtr,
                                   boolean cMemoryOwn)
      ```
  - ### Method Detail


    - #### activateMultilign

      ```
      public void activateMultilign()
      ```


    - #### activateTurboFold

      ```
      public void activateTurboFold()
      ```


    - #### addMultilignTuple

      ```
      public void addMultilignTuple(String seqFile,
                           String ctFile)
      ```


    - #### addTurboFoldTuple

      ```
      public void addTurboFoldTuple(String seqFile,
                           String ctFile)
      ```


    - #### buildAllSubDataStructure

      ```
      public String buildAllSubDataStructure(String file,
                                    boolean isRNA)
      ```


    - #### buildBifoldDataStructure

      ```
      public String buildBifoldDataStructure(String file1,
                                    String file2,
                                    boolean isRNA)
      ```


    - #### buildBipartitionDataStructure

      ```
      public String buildBipartitionDataStructure(String file1,
                                         String file2,
                                         boolean isRNA)
      ```


    - #### buildDynalignDataStructure

      ```
      public String buildDynalignDataStructure(String file1,
                                      String file2,
                                      boolean isRNA)
      ```


    - #### buildEfn2DataStructure

      ```
      public String buildEfn2DataStructure(String file,
                                  boolean isRNA)
      ```


    - #### buildFoldDataStructure

      ```
      public String buildFoldDataStructure(String file,
                                  boolean isRNA)
      ```


    - #### buildMaxExpectDataStructure

      ```
      public String buildMaxExpectDataStructure(String file)
      ```


    - #### buildOligoScreenDataStructure

      ```
      public String buildOligoScreenDataStructure()
      ```


    - #### buildOligoWalkDataStructure

      ```
      public String buildOligoWalkDataStructure(String file,
                                       boolean isRNA)
      ```


    - #### buildPartitionDataStructure

      ```
      public String buildPartitionDataStructure(String file,
                                       boolean isRNA)
      ```


    - #### buildProbKnotDataStructure

      ```
      public String buildProbKnotDataStructure(String file)
      ```


    - #### buildRefoldDynalignDataStructure

      ```
      public String buildRefoldDynalignDataStructure(String file)
      ```


    - #### buildRefoldSingleDataStructure

      ```
      public String buildRefoldSingleDataStructure(String file)
      ```


    - #### buildRemovePseudoknotsDataStructure

      ```
      public String buildRemovePseudoknotsDataStructure(String file,
                                               boolean isRNA)
      ```


    - #### buildStochasticDataStructure

      ```
      public String buildStochasticDataStructure(String file)
      ```


    - #### canFoldOligoOligo

      ```
      public boolean canFoldOligoOligo(int index)
      ```


    - #### canFoldOligoSelf

      ```
      public boolean canFoldOligoSelf(int index)
      ```


    - #### clearDynalignAlignmentConstraints

      ```
      public void clearDynalignAlignmentConstraints()
      ```


    - #### clearFoldingConstraints

      ```
      public void clearFoldingConstraints(int strand)
      ```


    - #### delete

      ```
      public void delete()
      ```


    - #### deleteMultilignTuple

      ```
      public void deleteMultilignTuple(long index)
      ```


    - #### deleteTurboFoldTuple

      ```
      public void deleteTurboFoldTuple(long index)
      ```


    - #### determineOligoMaximum

      ```
      public int determineOligoMaximum(int length)
      ```


    - #### finalize

      ```
      protected void finalize()
      ```

      **Overrides:**
      :   `finalize` in class `Object`


    - #### foldOligo

      ```
      public void foldOligo(String sequence,
                   int index,
                   boolean bimolecular,
                   boolean isRNA,
                   String file)
      ```


    - #### getAllOligoData

      ```
      public String getAllOligoData(int height)
      ```


    - #### getCPtr

      ```
      protected static long getCPtr(RNAstructureBackendCalculator obj)
      ```


    - #### getDisplayedOligo

      ```
      public String getDisplayedOligo(int index)
      ```


    - #### getDynalignAlignmentConstraints

      ```
      public String getDynalignAlignmentConstraints()
      ```


    - #### getDynalignAlignmentWindowSize

      ```
      public int getDynalignAlignmentWindowSize()
      ```


    - #### getDynalignStructureWindowSize

      ```
      public int getDynalignStructureWindowSize()
      ```


    - #### getFoldingConstraints

      ```
      public String getFoldingConstraints(int strand)
      ```


    - #### getFoldWindowSize

      ```
      public int getFoldWindowSize()
      ```


    - #### getGraphRegionBegin

      ```
      public int getGraphRegionBegin()
      ```


    - #### getGraphRegionEnd

      ```
      public int getGraphRegionEnd()
      ```


    - #### getMaxConstraintIndex

      ```
      public int getMaxConstraintIndex(int strand)
      ```


    - #### getMaxLoop

      ```
      public int getMaxLoop()
      ```


    - #### getMaxPair

      ```
      public int getMaxPair()
      ```


    - #### getMostStableOligo

      ```
      public int getMostStableOligo()
      ```


    - #### getMultilignCT

      ```
      public String getMultilignCT(int index)
      ```


    - #### getMultilignMaxPairs

      ```
      public int getMultilignMaxPairs()
      ```


    - #### getMultilignSequenceSetData

      ```
      public String getMultilignSequenceSetData()
      ```


    - #### getNumMultilignSequences

      ```
      public int getNumMultilignSequences()
      ```


    - #### getNumTurboFoldSequences

      ```
      public int getNumTurboFoldSequences()
      ```


    - #### getOligoLabelData

      ```
      public String getOligoLabelData(int index)
      ```


    - #### getOligoTargetLength

      ```
      public int getOligoTargetLength()
      ```


    - #### getOligoTargetSequence

      ```
      public String getOligoTargetSequence()
      ```


    - #### getProgressNumber

      ```
      public int getProgressNumber()
      ```


    - #### getRefoldWindowSize

      ```
      public int getRefoldWindowSize()
      ```


    - #### getSequenceComment

      ```
      public String getSequenceComment()
      ```


    - #### getSequenceData

      ```
      public String getSequenceData()
      ```


    - #### getSequenceTitle

      ```
      public String getSequenceTitle()
      ```


    - #### getStructureType

      ```
      public String getStructureType()
      ```


    - #### getSuboptimalAbsoluteDiff

      ```
      public double getSuboptimalAbsoluteDiff()
      ```


    - #### getSuboptimalPercentDiff

      ```
      public float getSuboptimalPercentDiff()
      ```


    - #### getTemperature

      ```
      public double getTemperature()
      ```


    - #### getTurboFoldCT

      ```
      public String getTurboFoldCT(int index)
      ```


    - #### getTurboFoldSaveFile

      ```
      public String getTurboFoldSaveFile(int index)
      ```


    - #### getTurboFoldSequenceSetData

      ```
      public String getTurboFoldSequenceSetData()
      ```


    - #### readDynalignAlignmentConstraintsFile

      ```
      public String readDynalignAlignmentConstraintsFile(String file)
      ```


    - #### readFoldingConstraintsFile

      ```
      public String readFoldingConstraintsFile(String file,
                                      int strand)
      ```


    - #### readSequenceData

      ```
      public void readSequenceData(String file)
      ```


    - #### runAllSub

      ```
      public String runAllSub(String ct,
                     float percent,
                     double absolute)
      ```


    - #### runBifold

      ```
      public String runBifold(String ctFile,
                     float percent,
                     int maxStructures,
                     int windowSize,
                     boolean saveFile,
                     boolean intraForbidden)
      ```


    - #### runBipartition

      ```
      public String runBipartition(String pfsFile)
      ```


    - #### runDynalign

      ```
      public String runDynalign(String ctFile1,
                       String ctFile2,
                       String saveFile,
                       String alignFile,
                       double percent,
                       int structures,
                       int windowStr,
                       int windowAli,
                       float gap,
                       boolean isInsert)
      ```


    - #### runDynalignRefold

      ```
      public String runDynalignRefold(String ctFile1,
                             String ctFile2,
                             String alignFile,
                             int percent,
                             int structures,
                             int windowStr,
                             int windowAli)
      ```


    - #### runEfn2

      ```
      public String runEfn2(String outFile,
                   boolean writeDetails)
      ```


    - #### runFold

      ```
      public String runFold(String ctFile,
                   int percent,
                   int structures,
                   int window,
                   boolean save)
      ```


    - #### runMaxExpect

      ```
      public String runMaxExpect(String ctFile,
                        double score,
                        int structures,
                        int window,
                        double gamma)
      ```


    - #### runMultilign

      ```
      public String runMultilign(int percent,
                        int maxStructures,
                        int bpWindow,
                        int alignWindow,
                        double gap,
                        boolean insert,
                        double maxDsvChange,
                        int maxPairs,
                        int cycles,
                        String alignFile,
                        boolean saveFiles,
                        boolean isRNA)
      ```


    - #### runOligoScreen

      ```
      public String runOligoScreen(String in,
                          String out,
                          boolean isRNA)
      ```


    - #### runOligoWalk

      ```
      public String runOligoWalk(String report,
                        int mode,
                        String chemistry,
                        boolean suboptimal,
                        int length,
                        int amount,
                        String unit,
                        int start,
                        int stop)
      ```


    - #### runPartition

      ```
      public String runPartition(String pfsFile)
      ```


    - #### runPseudoknotPrediction

      ```
      public String runPseudoknotPrediction(String ctFile,
                                   int iterations,
                                   int helix)
      ```


    - #### runPseudoknotRemoval

      ```
      public String runPseudoknotRemoval(String ctFile,
                                boolean minimize)
      ```


    - #### runRefold

      ```
      public String runRefold(String ctFile,
                     int percent,
                     int structures,
                     int window)
      ```


    - #### runStochastic

      ```
      public String runStochastic(String outFile,
                         int ensemble,
                         int seed)
      ```


    - #### runTurboFoldMaximumExpectedAccuracy

      ```
      public String runTurboFoldMaximumExpectedAccuracy(double turboGamma,
                                               int turboIterations,
                                               double percent,
                                               int structures,
                                               int window,
                                               double meaGamma)
      ```


    - #### runTurboFoldPseudoknot

      ```
      public String runTurboFoldPseudoknot(double turboGamma,
                                  int turboIterations,
                                  int pkIterations,
                                  int helix)
      ```


    - #### runTurboFoldThreshold

      ```
      public String runTurboFoldThreshold(double turboGamma,
                                 int turboIterations,
                                 double threshold)
      ```


    - #### setCleavedNucleotide

      ```
      public String setCleavedNucleotide(int nuc,
                                int strand)
      ```


    - #### setDoubleStrandedNucleotide

      ```
      public String setDoubleStrandedNucleotide(int nuc,
                                       int strand)
      ```


    - #### setDynalignAlignmentConstraint

      ```
      public String setDynalignAlignmentConstraint(int nuc1,
                                          int nuc2)
      ```


    - #### setForcedHelix

      ```
      public String setForcedHelix(int nuc1,
                          int nuc2,
                          int length,
                          int strand)
      ```


    - #### setMaxLoop

      ```
      public void setMaxLoop(int newLoop)
      ```


    - #### setMaxPair

      ```
      public void setMaxPair(int newPair)
      ```


    - #### setModifiedNucleotide

      ```
      public String setModifiedNucleotide(int nuc,
                                 int strand)
      ```


    - #### setProhibitedHelix

      ```
      public String setProhibitedHelix(int nuc1,
                              int nuc2,
                              int length,
                              int strand)
      ```


    - #### setSequenceComment

      ```
      public void setSequenceComment(String comment)
      ```


    - #### setSequenceData

      ```
      public void setSequenceData(String data)
      ```


    - #### setSequenceTitle

      ```
      public void setSequenceTitle(String title)
      ```


    - #### setSHAPEFile

      ```
      public void setSHAPEFile(String file)
      ```


    - #### setSHAPEParam1

      ```
      public void setSHAPEParam1(double value)
      ```


    - #### setSHAPEParam2

      ```
      public void setSHAPEParam2(double value)
      ```


    - #### setSHAPEType

      ```
      public void setSHAPEType(boolean isEnergy)
      ```


    - #### setSingleStrandedNucleotide

      ```
      public String setSingleStrandedNucleotide(int nuc,
                                       int strand)
      ```


    - #### setTemperature

      ```
      public void setTemperature(double newTemp)
      ```


    - #### writeDynalignAlignmentConstraintsFile

      ```
      public void writeDynalignAlignmentConstraintsFile(String file)
      ```


    - #### writeFastaFile

      ```
      public String writeFastaFile(String file)
      ```


    - #### writeFoldingConstraintsFile

      ```
      public String writeFoldingConstraintsFile(String file,
                                       int strand)
      ```


    - #### writeSequenceFile

      ```
      public String writeSequenceFile(String file)
      ```


- Overview
- Package
- Class
- Tree
- Index
- Help

- Prev Class
- Next Class

- Frames
- No Frames

- All Classes

- Summary:
- Nested |
- Field |
- Constr |
- Method

- Detail:
- Field |
- Constr |
- Method
